# Supplementary material for: Venomix: a simple bioinformatic pipeline for identifying and characterizing toxin gene candidates from transcriptomic data
Source: PeerJ. 2018 Jul 31;6:e5361. doi: 10.7717/peerj.5361 (PMC6074769; doi:10.7717/peerj.5361)
Supplement: Supplemental Information 6 [file peerj-06-5361-s006.gz › FinalOutput_1E-6/Venom_allergen_5_2_1/finaltree.pdf]

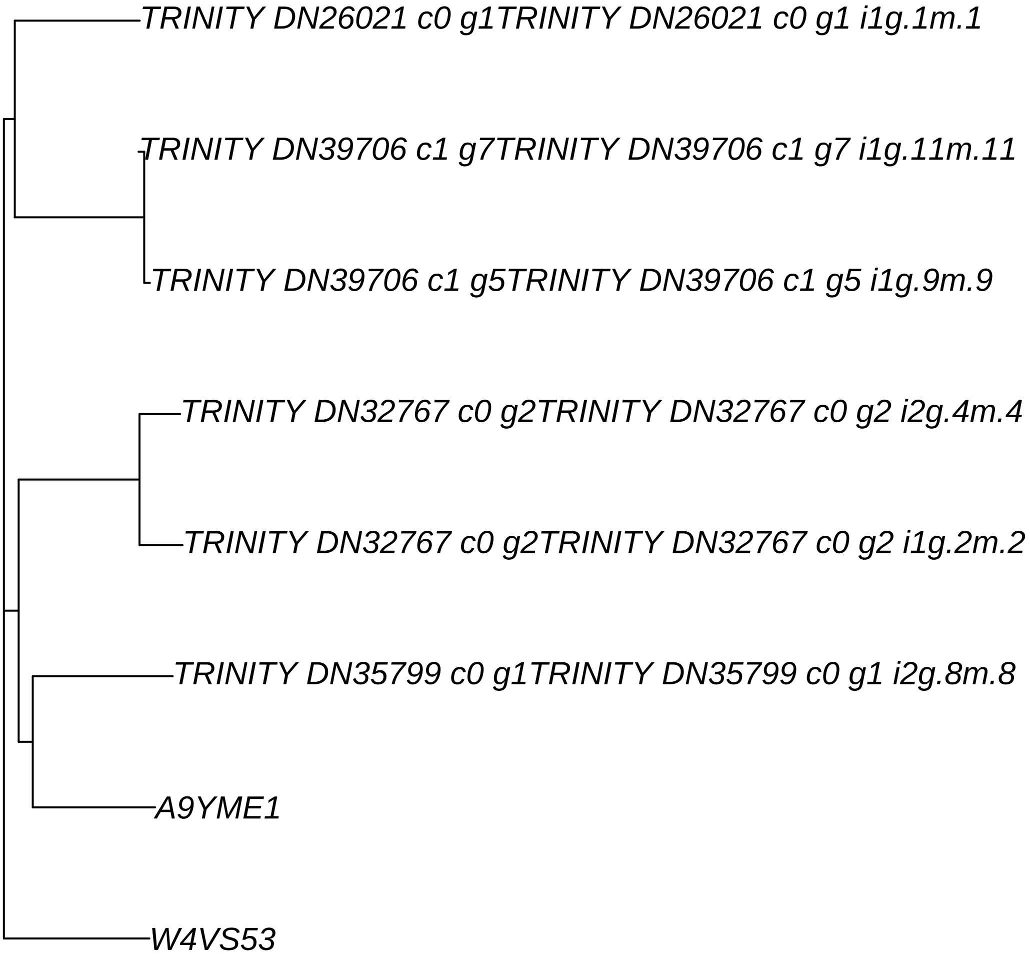
TRINITY DN26021 c0 g1TRINITY DN26021 c0 g1 i1g.1m.1
TRINITY DN39706 c1 g7TRINITY DN39706 c1 g7 i1g.11m.11
TRINITY DN39706 c1 g5TRINITY DN39706 c1 g5 i1g.9m.9
TRINITY DN32767 c0 g2TRINITY DN32767 c0 g2 i2g.4m.4
TRINITY DN32767 c0 g2TRINITY DN32767 c0 g2 i1g.2m.2
TRINITY DN35799 c0 g1TRINITY DN35799 c0 g1 i2g.8m.8
A9YME1
W4VS53
